# Abdominal computed tomography scoring systems and experienced radiologists in the radiological diagnosis of small bowel and mesenteric injury

**DOI:** 10.1007/s10140-023-02197-8

**Published:** 2024-02-20

**Authors:** Devin M. O’Toole, Nicole V. Warrington, Nicholas G Matthees, Kristina M. Kupanoff, James N. Bogert, Michael D. Jones, Hahn Soe-Lin, Dih-Dih Huang, Jordan A. Weinberg

**Affiliations:** 1 Creighton University School of Medicine, Phoenix Campus, Phoenix, AZ USA; 2 Creighton University Arizona Health Education Alliance, Phoenix, AZ USA; 3 Barrow Neurological Institute, Phoenix, AZ USA; 4 Trauma/Acute General Surgery, Trauma Administration, St. Joseph’s Hospital and Medical Center, 350 W. Thomas Road, Phoenix, AZ 85013 USA

**Keywords:** Trauma, Blunt bowel injury, Radiologist interpretation, Injury prediction, RAPTOR, BIPS

## Abstract

**Purpose:**

Blunt bowel and/or mesenteric injury requiring surgery presents a diagnostic challenge. Although computed tomography (CT) imaging is standard following blunt trauma, findings can be nonspecific. Most studies have focused on the diagnostic value of CT findings in identifying significant bowel and/or mesenteric injury (sBMI). Some studies have described scoring systems to assist with diagnosis. Little attention, has been given to radiologist interpretation of CT scans. This study compared the discriminative ability of scoring systems (BIPS and RAPTOR) with radiologist interpretation in identifying sBMI.

**Methods:**

We conducted a retrospective chart review of trauma patients with suspected sBMI. CT images were reviewed in a blinded fashion to calculate BIPS and RAPTOR scores. Sensitivity and specificity were compared between BIPS, RAPTOR, and the admission CT report with respect to identifying sBMI.

**Results:**

One hundred sixty-two patients were identified, 72 (44%) underwent laparotomy and 43 (26.5%) had sBMI. Sensitivity and specificity were: BIPS 49% and 87%, AUC 0.75 (0.67–0.81), *P* < 0.001; RAPTOR 46% and 82%, AUC 0.72 (0.64–0.79), *P* < 0.001; radiologist impression 81% and 71%, AUC 0.82(0.75–0.87), *P* < 0.001. The discriminative ability of the radiologist impression was higher than RAPTOR (*P* = 0.04) but not BIPS (*P* = 0.13). There was not a difference between RAPTOR vs. BIPS (*P* = 0.55).

**Conclusion:**

Radiologist interpretation of the admission CT scan was discriminative of sBMI. Although surgical vigilance, including evaluation of the CT images and patient, remains fundamental to early diagnosis, the radiologist’s impression of the CT scan can be used in clinical practice to simplify the approach to patients with abdominal trauma.

## Introduction

Bowel and/or mesenteric injury requiring operation following blunt trauma presents a diagnostic challenge because it occurs infrequently and can present latently [1–4]. Computed tomography (CT) imaging of the abdomen/pelvis is commonly used to identify significant blunt bowel and/or mesenteric injury (i.e., those injuries requiring operative repair), but findings are often nonspecific and many patients with imaging findings that are coded as bowel and/or mesenteric injuries do not require intervention (i.e., insignificant bowel and/or mesenteric injury). The presence of particular CT findings including bowel discontinuity, extraluminal air, and mesenteric contrast extravasation are associated with significant bowel and/or mesenteric injury and require operative repair. Often, however, CT findings are less specific, leaving the surgeon with a diagnostic dilemma: to proceed with a possibly unnecessary intervention or to observe the patient, with the risk of delaying [5] a necessary intervention.

Prior studies have focused on the predictive value of specific CT findings, such as free intraperitoneal fluid, with respect to significant bowel and/or mesenteric injury [6–9]. Other groups have developed scoring systems to assist clinical decision-making [10–12]. One such scoring system is the radiographic predictors of therapeutic intervention (RAPTOR) score, which utilizes seven CT findings to generate an 8-point score (a score of 3 or more suggests significant bowel and/or mesenteric injury) [10]. The Bowel Injury Prediction Score (BIPS) utilizes CT, laboratory, and physical exam findings to generate a predictive score between 0 and 3 with a score of 2 or greater associated with significant injury [11]. A shared characteristic of these prior predictive studies is that they are generally agnostic to the fact that the CT scan has been interpreted by a radiologist and an overall impression of the imaging is typically included in the study report. To our knowledge, no study has determined the predictive ability of the radiologist interpretation of the CT scan in determining the presence of significant bowel and/or mesenteric injury. The purpose of this study was to compare the discriminative ability of two of the described scoring systems (BIPS and RAPTOR) with radiologist interpretation with respect to the identification of significant bowel and/or mesenteric injury. We hypothesized that the radiologist’s interpretation of the CT scan would predict significant bowel and/or mesenteric injury as equally well as BIPS and/or RAPTOR.

## Methods

This retrospective study received approval from our institution’s Internal Review Board. Adult patients admitted to our level 1 trauma center between June 2014 and September 2021 with blunt bowel and/or mesenteric injuries based on AIS-2005 discharge injury codes were included. Patients must have obtained a CT scan of the abdomen/pelvis at the time of admission (GE Lightspeed CT, Milwaukee, WI). CT scans were all obtained with intravenous contrast, scanned in the venous phase as well as 3-min delayed images through the kidneys and bladder. No oral contrast was given. Injury coding was performed by certified trauma registrars who used the medical record of each patient to determine injury codes. Bowel and mesenteric injuries specifically were coded from CT scan reports, along with operative reports for those patients who underwent laparotomy. Patients who expired during hospitalization without undergoing laparotomy were excluded from the analysis.

Significant bowel and/or mesenteric injury (sBMI) was confirmed if operative findings included a bleeding mesenteric injury requiring ligation and/or bowel injury requiring repair or resection. The radiologist’s impression of the admission CT abdomen/pelvis scan was reviewed for each patient and given a score from 0 to 2 according to the likelihood of sBMI (0 signifying no or unlikely SBMI, 1 signifying possible sBMI, 2 signifying high likelihood of sBMI). The reviewer for sBMI was a medical student, mentored by the senior surgeon on the study. The student reviewed the radiologist’s conclusions for each scan and identified terminology that assessed the probability of bowel injury. The words “no evidence of” were used for all scans classified as no evidence of bowel injury. Terms such as “consistent with” or “concerning for” were used for scans classified as “high likelihood” and scans with diagnostic descriptions with terms such as “cannot rule out” and “possible” were classified as possible bowel injury. The senior author audited the student’s scoring of the initial 15 scans reviewed and then adjudicated on any interpretation the student requested assistance. The patients who did not undergo laparotomy during hospitalization or underwent laparotomy that was nontherapeutic, meaning exploratory and without repair, were considered to have had insignificant bowel and/or mesenteric injury.

One attending level radiologist and one senior resident radiologist reviewed CT images for each patient. Admission CT scans were reviewed to assign a RAPTOR score as described by Filiberto et al. and the CT interpretation component of the BIPS score as described by McNutt et al. [10, 11]. The radiologists were blinded to the original CT scan report and the hospital course of the patient. The additional components of the BIPS score (leukocyte count, presence or absence of abdominal tenderness) were determined by medical record review. The grading criteria for the three scales are shown in Table 1. RAPTOR scores range from 0 to 7, BIPS scores range from 0 to 3, and radiologist interpretations ranged from 0 to 2, all with higher scores indicating increasing injury severity.

**Table 1:** Scoring

RAPTOR	Feature	Points
	Multifocal hematoma	1
	Acute arterial extravasation	1
	Bowel wall hematoma	1
	Devascularization,	1
	Fecalization	1
	Pneumoperitoneum	1
	Fat pad injury	1
	Total possible RAPTOR score	7
BIPS	Feature	Points
	White blood cell count of ≥17 g/l	1
	Abdominal tenderness	1
	CT grade 4 or 5	1
	Total possible BIPS score	3
BIPS: CT Grade	Feature	Grade
	Isolated mesenteric contusion without associated BWT or adjacent interloop fluid collection	1
	Mesenteric hematoma < 5 cm without associated BWT or adjacent interloop fluid collection	2
	Mesenteric hematoma > 5 cm without associated BWT or adjacent interloop fluid collection	3
	Mesenteric contusion or hematoma (any size) with associated bowel wall thickening or adjacent interloop fluid collection	4
	Active vascular or oral contrast extravasation, bowel transection, or pneumoperitoneum	5
Radiologist determination	Likelihood of bowel injury	Score
	No evidence	0
	Possible	1
	High likelihood	2

Abbreviations: *BIPS* Bowel Injury Prediction Score, *BWT* bowel wall thickening, *CT* computed tomography, *RAPTOR* radiographic predictors of therapeutic intervention

The Injury Severity Score (ISS) was categorized into three groups as standardly reported, <9, 9–15, and 16+. The length of stay was a continuous variable with medians reported due to non-normality. BIPS, RAPTOR, and radiologist impressions were considered both continuously and as a dichotomized variable. The cohort was described using counts with percentages, means with standard deviations, or medians with interquartile ranges as appropriate. 2 × 2 comparisons were made using Pearson chi-square tests or Fisher’s exact test given an expected cell count <5. Receiver operating characteristic (ROC) curves were used to compare the 3 measures as predictors of sBMI. The area under the curve (AUC) was calculated for each measure and the difference in AUCs was compared using the z-statistic. There were no missing data. Statistical analyses were performed using SPSS version 27 (IBM Corp, Armonk, NY, USA).

## Results

A total of 262 trauma patients admitted between June 2014 and September 2021 with bowel and/or mesenteric injuries were identified in our institution’s trauma registry which consisted of 12,336 patients admitted to or consulted by our trauma service. Exclusions included one pediatric patient, 92 patients who lacked an admission abdominal CT scan, and seven patients who expired in hospital without laparotomy, leaving a total of 162 patients in our cohort. These seven patients all underwent palliative care for severe traumatic brain injury.

The 162 patients in our cohort were on average 40.9 ± 17.6 years of age, the majority male (69.1%) and White (54.9%) (Table 2). The most common mechanism of injury was motor vehicle crash (81.5%) with 65.4% of patients having an ISS of 16 or greater. Overall median length of stay was 7 (4 – 13) days. Seventy-two patients underwent laparotomy and 43 (59.7%) had sBMI, leaving 29 (40.3%) who underwent non-therapeutic surgery. The average length of stay for these patients was 9.5(5.0 – 15.5) days. 90 patients did not undergo laparotomy and had a median length of stay of 5.5(3.0 – 12.0) days.

**Table 2:** Patient and injury characteristics

	Cohort (*n* = 162)
Age	40.9 ± 17.6
Sex, male	112 (69.1%)
Race	
American Indian/Alaska Native	6 (3.7%)
Asian	3 (1.9%)
Black	7 (4.3%)
Other White	57 (35.2%)
	89 (54.9%)
Ethnicity, Hispanic	62 (38.3%)
Mechanism of injury	
Fall	15 (9.3%)
MVA	132 (81.5%)
Other	5 (3.1%)
Pedacyclist	2 (1.2%)
Struck by or against	8 (4.9%)
Injury Severity Score	
< 9	21 (13.0%)
9–15	35 (21.6%)
16+	106 (65.4%)
Intensive care unit admission	140 (86.4%)
Hospital disposition	
Left against medical advice	3 (1.9%)
Skilled nursing facility	49 (30.2%)
Expired	14 (8.6%)
Home	75 (46.3%)
Intermediate or long-term care	5 (3.1%)
Psychiatric	1 (0.6%)
Rehabilitation	15 (9.3%)

Each BIPS and RAPTOR scoring feature is listed in Table 3 with the corresponding count and percentage present with and without sBMI. The presence of two of the three BIPS criteria (abdominal tenderness and CT grade > 3) was more prevalent in the group with sBMI (60.5% vs 34.5%, *P* =.003 and 65.1% vs 18.5%, *P* < .001). WBC > 17 g/l did not discriminate between patients with or without sBMI (*P* = 0.670). Five of the 7 RAPTOR criteria (multifocal hematoma, acute arterial extravasation, bowel wall hematoma, pneumoperitoneum, and fat pad injury) were more prevalent in sBMI groups (Table 3).

**Table 3:** Comparison of BIPS and RAPTOR score features with bowel and/or mesenteric injury

	Insignificant bowel or mesenteric injury (*n* = 119)	Significant bowel or mesenteric injury (*n* = 43)	OR (95% CI)	*p*-value
Predicting blunt bowel injury				
BIPS criteria				
WBC > 17	24 (20.2%)	10 (23.3%)	1.2 (0.5 – 2.8)	0.670
Abdominal tenderness	41 (34.5%)	26 (60.5%)	2.9 (1.4 – 6.0)	0.003
CT Grade 4 or 5	22 (18.5%)	28 (65.1%)	8.2 (3.8 – 17.9)	<0.001
RAPTOR criteria				
Multifocal hematoma	6 (5.0%)	14 (32.6%)	9.1 (3.2 – 25.7)	<0.001
Acute arterial extravasation	7 (5.9%)	11(25.6%)	5.5 (2.0 – 15.3)	0.001
Bowel wall hematoma	6 (5.0%)	8 (18.6%)	4.3 (1.4 – 13.3)	0.011
Bowel devascularization	0	1 (2.3%)	--	0.265
Fecalization	22 (18.5%)	10 (23.3%)	1.3 (0.6 – 3.1)	0.501
Pneumoperitoneum	0	7 (16.3%)	--	<0.001
Fatpad injury	43 (36.1%)	24 (55.8%)	2.2 (1.1 – 4.5)	0.025

Abbreviations: *BIPS* Bowel Injury Prediction Score, *CT* computed tomography, *OR* odds ratio, *RAPTOR* radiographic predictors of therapeutic intervention, *WBC* white blood cells

The distribution of BIPS, RAPTOR, and radiologist impression scores is shown in Table 4. Seventy-five percent of scores were either 0 or 1 for all three scales. 3.7%, 1.2%, and 14.2% of cases were categorized as the most severe score by BIPS, RAPTOR, and radiologist impression. As shown in Table 4, radiologist impression was no or unlikely sBMI on 99 of the 162 scans. However, 7 (7.1%) of these patients had sBMI as confirmed by laparotomy. Sixteen (40%) of the 40 scans determined by radiologists to have a possible sBMI were confirmed by laparotomy, and 20 (87%) of the high-likelihood cases had sBMI confirmed by laparotomy.

**Table 4:** Distribution of BIPS, RAPTOR, and radiologist impression

Score	BIPS	RAPTOR	Radiologist impression
0	54 (33.3%)	64 (39.5%)	99 (61.1%)
1	71 (43.8%)	57 (35.2%)	40 (24.7%)
2	31 (19.1%)	26 (16.0%)	23 (14.2%)
3	6 (3.7%)	11 (6.8%)	N/A
4	N/A	2 (1.2%)	N/A
5+	N/A	2 (1.2%)	N/A

Abbreviations: *BIPS* Bowel Injury Prediction Score, *RAPTOR* radiographic predictors of therapeutic intervention

**Fig. 1:**
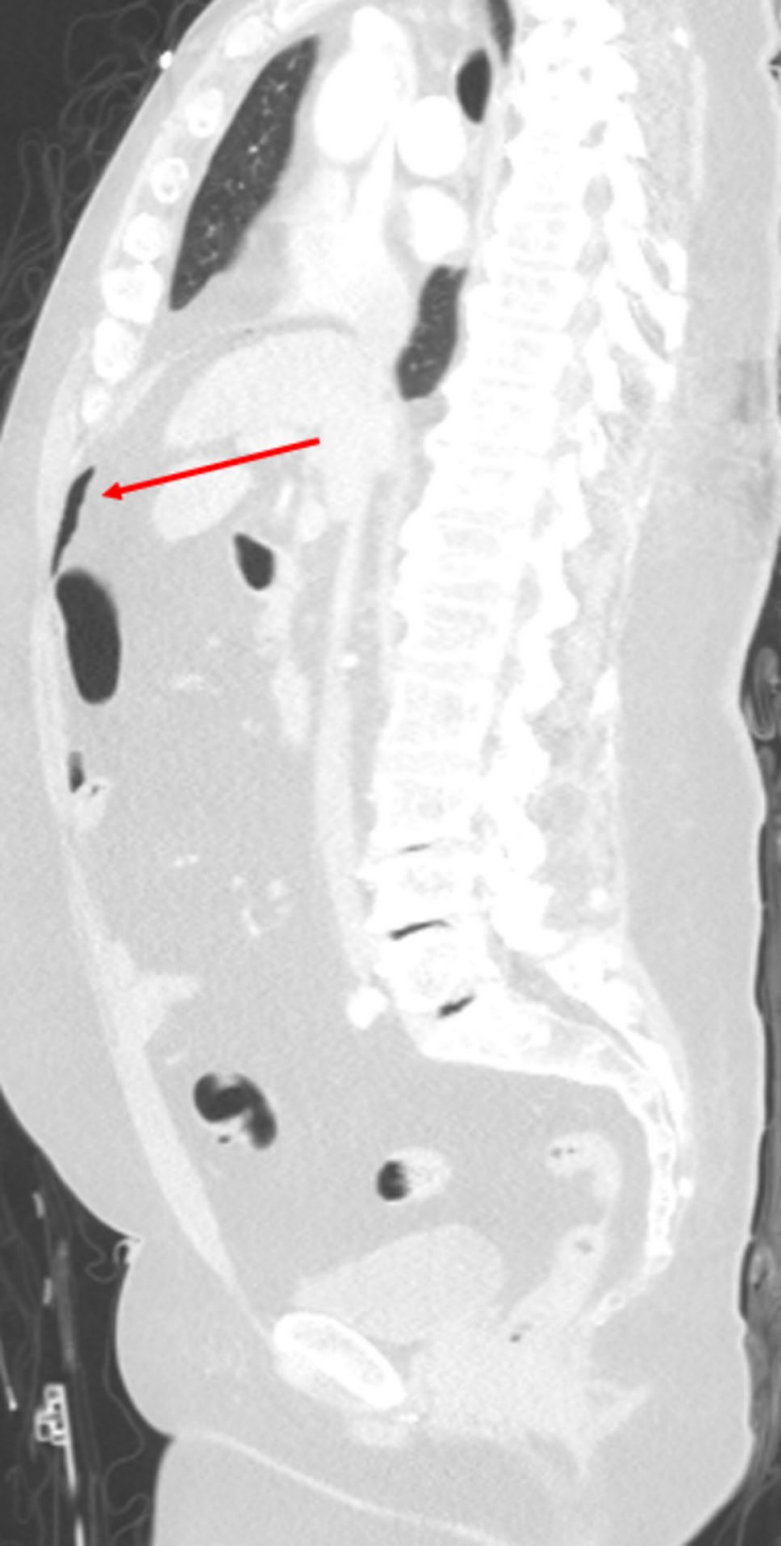
Sagittal CT in lung window demonstrates a large amount of free air (red arrow). There were no other signs of bowel injury according to the RAPTOR grading; however, the patient was confirmed to have an injury of the distal ileum. This was graded as a 2 given the large volume of free air

**Fig. 2:**
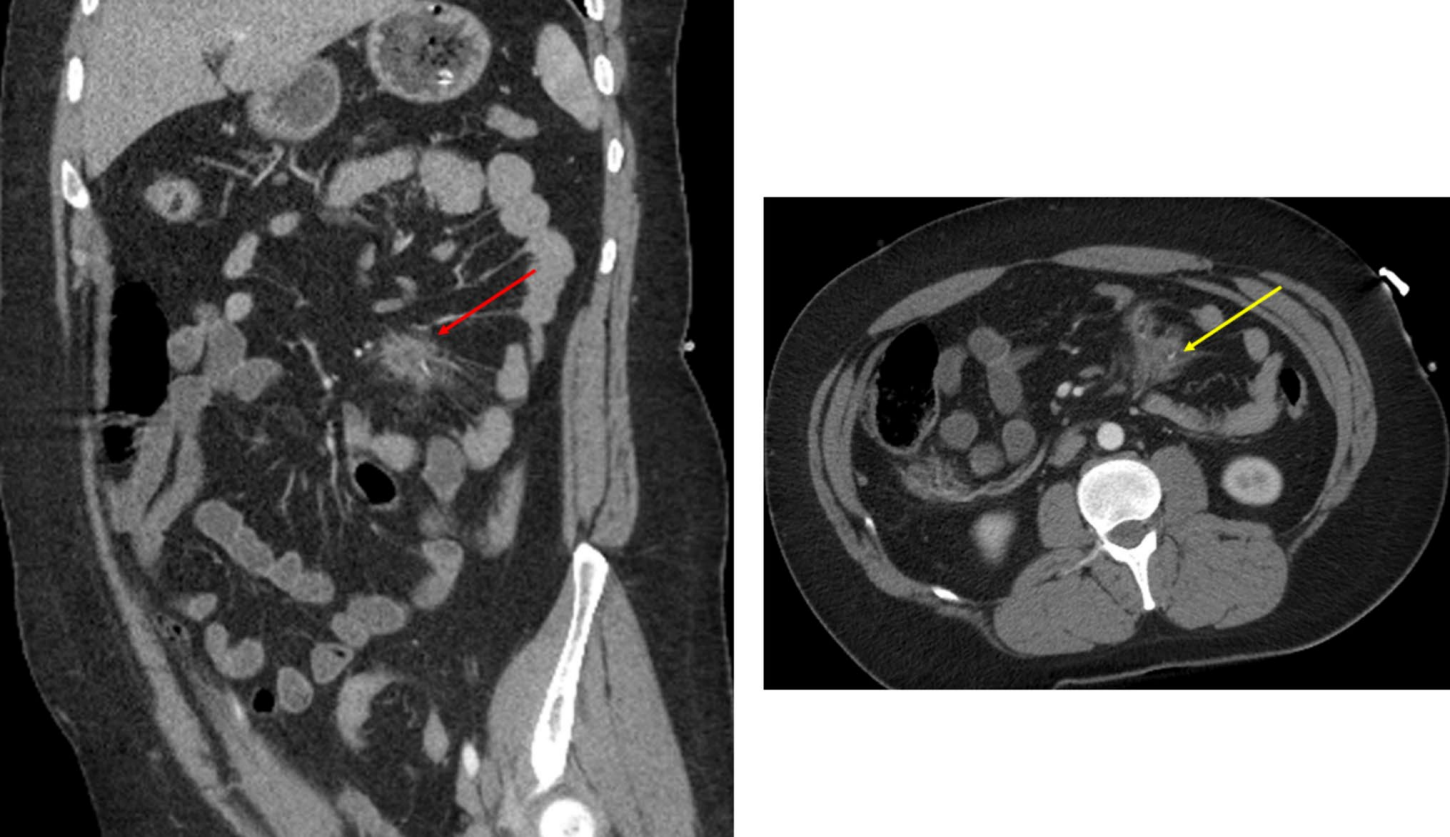
(Left) Coronal CT of the abdomen demonstrates a small mesenteric contusion (red arrow). (Right) Axial CT shows the mesenteric contusion with a small focus of active extravasation (yellow arrow). This patient had no bowel injury upon exploratory laparotomy

**Fig. 3:**
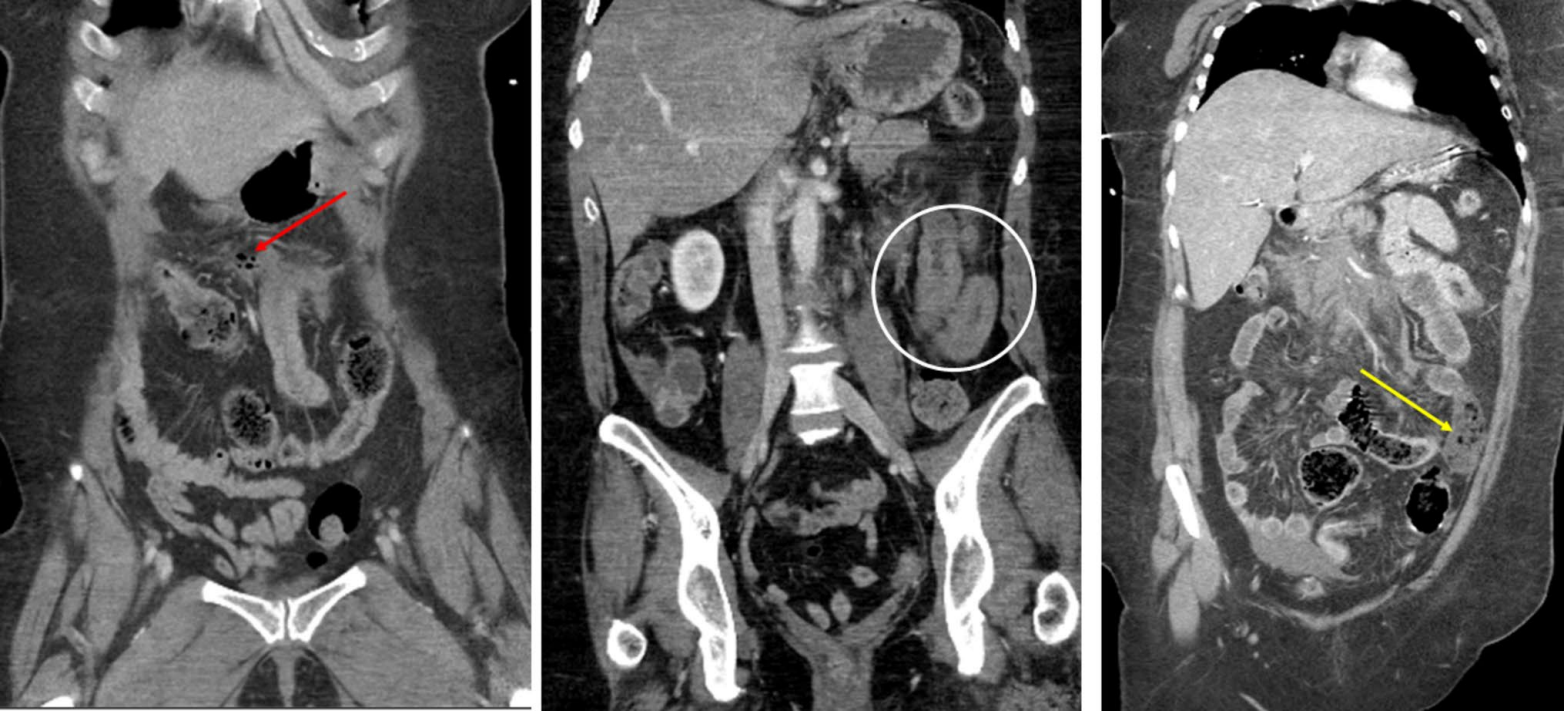
(Left) Coronal CT of the abdomen showing a collection of free air and fluid adjacent to a small bowel loop (red arrow). (Center) Coronal CT of the abdomen in a different patient showing bowel wall thickening with interloop fluid collection (white circle). (Right) Coronal CT of the abdomen in a different patient showing a devascularized bowel (yellow arrow)

Figure 1 demonstrates a patient with a suspected high likelihood of injury via radiology impression (score = 2) and BIPS (BIPS CT Grade = 5), but a low likelihood on RAPTOR (score = 1). This patient had a confirmed small bowel injury. Figure 2 demonstrates a mesenteric contusion with active bleeding and had a BIPS score of 5 and a RAPTOR score of 2 and a radiologist impression score of 0. This patient did not have a confirmed bowel injury. Examples of radiologic findings of patients with confirmed bowel injuries are shown in Fig. 3.

**Fig. 4:**
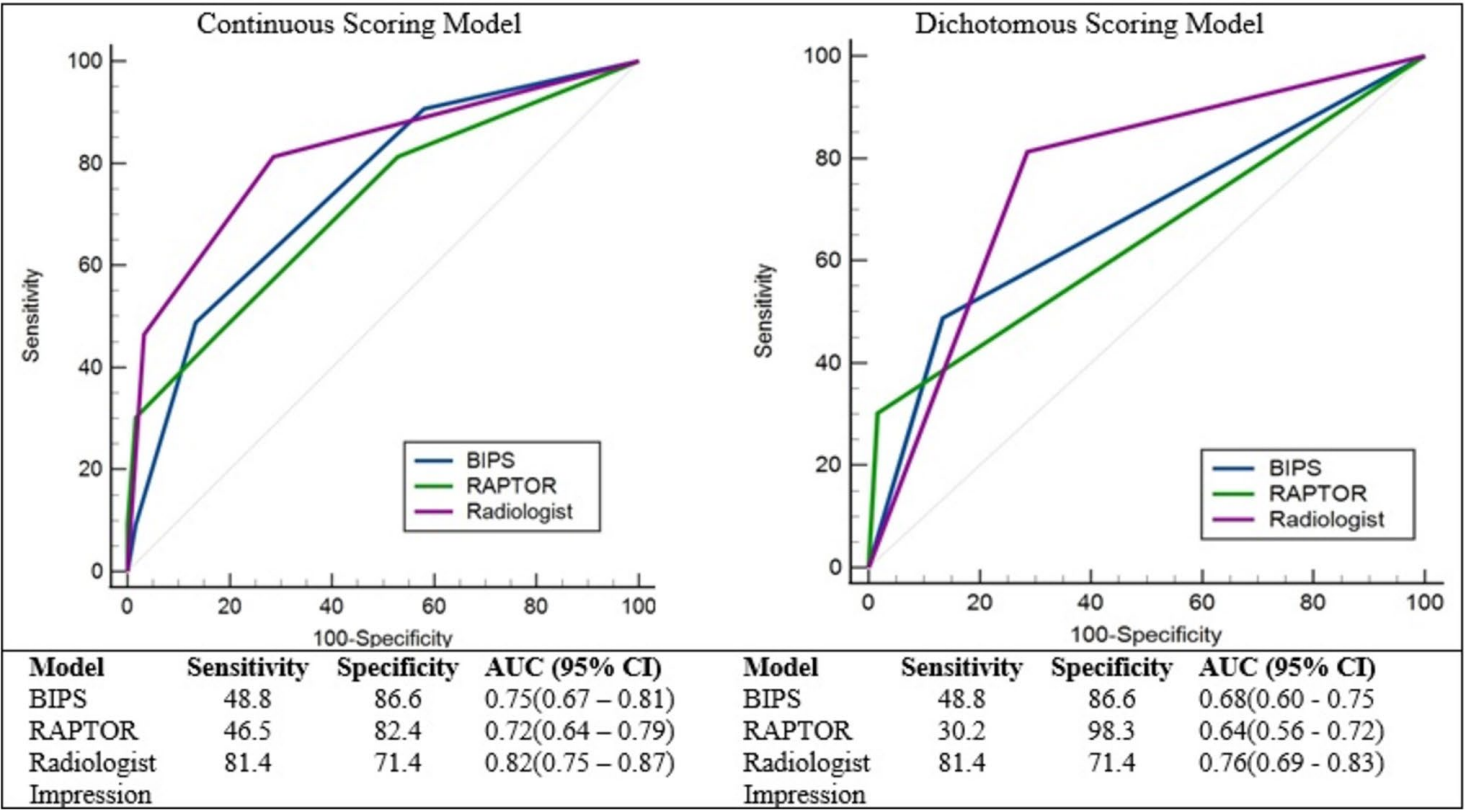
Receiver operating characteristic curves for BIPS, RAPTOR, and radiologist impression in predicting blunt bowel and/or mesenteric injury. Abbreviations: AUC, area under the curve; BIPS, Bowel Injury Prediction Score; CI, confidence interval; CT, computed tomography; RAPTOR, radiographic predictors of therapeutic intervention

Sensitivity and specificity with the area under the curve are shown in Fig. 4. BIPS and RAPTOR were highly specific (86.6% and 82.4%) but not sensitive (48.8% and 46.5%) with AUCs of 0.75 and 0.72 (Fig. 4, left). Radiologist impression exceeded the sensitivity of the others at 81.4% with only a slight drop in specificity (71.4%) and the overall highest AUC of 0.82. There was not a significant difference between BIPS and RAPTOR AUCs (*P* = 0.55). Radiologist impression was the superior discriminator of sBMI when compared to RAPTOR (*P* = 0.04) but not BIPS (*P* = 0.13).

Thus far, the AUCs presented are based on the continuous score of BIPS, RAPTOR, and radiologist impression. The creators of the BIPS and RAPTOR scores determined that threshold scores of 2 and 3 for BIPS and RAPTOR respectively most accurately predict sBMI. In this study, 25% (40/162) of radiologist impressions were categorized as possible sBMI, and 40% of these were found to have sBMI. Thus, we grouped the possible sBMI and high likelihood sBMI cases together and repeated our analysis using the dichotomous scale (i.e., score of zero vs. ≥ 1). Thresholds of > 3 for RAPTOR and >2 for BIPS were used for this analysis. As expected, the AUCs for each measure dropped; however, radiologist impression remained associated with the highest AUC (0.76) followed by BIPS (0.68) and then RAPTOR (0.64) (Fig. 4, right).

## Discussion

Many studies have investigated the association between specific CT findings and confirmed sBMI at laparotomy, such as free fluid or free air [7–9]. To assist further in the diagnosis of sBMI, rubrics that account for multiple CT findings by utilizing scoring systems have been proposed, including the RAPTOR score that was evaluated in the current study. Other groups have included laboratory and physical exam findings in scoring systems that attempt to further improve the accuracy of diagnosis, including the BIPS score that was also evaluated in the current study. In this study, we evaluated the radiologist interpretation of the initial CT scans performed in the setting of blunt trauma, and we identified that the radiologist’s impression has relatively highly sensitive and reasonably specificity for detecting sBMI as determined by the patient’s subsequent hospital course.

Accurate and rapid determination of sBMI is one of the more difficult diagnostic challenges faced by the trauma surgeon. Delayed diagnosis of sBMI carries with it a real risk of morbidity and mortality [13, 14], but operative evaluation for every incidental abdominal CT finding would result in an unacceptable rate of nontherapeutic laparotomy. It is not surprising then that there are many studies that have evaluated the relative predictive value of specific CT findings or have proposed scoring systems that account for constellations of CT findings or the combination of imaging results with clinical and laboratory findings. However, prior studies have not directly assessed the likelihood of sBMI based on the radiologist’s overall impression of the CT scan.

After stratifying our radiologists’ interpretations into no likelihood (zero points), possible sBMI (1 point), or high likelihood of sBMI (2 points), we determined that the AUC for the associated receiver operating characteristic curve was 0.82 which is relatively high. Nonetheless, this observation must be interpreted in the context of clinical decision-making. A high likelihood of sBMI was associated with operative corroboration 87% of the time, so it would be reasonable to perform exploratory laparotomy (or laparoscopy) in this setting. Conversely, no likelihood of sBMI was falsely negative only 7.1% of the time, so it would be reasonable to expect that the need for operative exploration in these patients will be unlikely. For those patients with a CT interpretation that is not definitive (i.e., possible sBMI), clinical decision-making remains difficult. In this study, 40% of these patients were proven to have sBMI. It is our opinion that for such patients, the surgeon should lean toward operative exploration by either laparotomy or laparoscopy, accepting a higher rate of nontherapeutic laparotomy at the expense of not delaying therapy for sBMI.

Although the radiologist interpretation of the CT scan slightly outperformed both scoring systems, the utility of both the BIPS and RAPTOR scores (or similar scoring systems like them) should not be entirely discounted. These scoring systems may still be helpful for trauma surgeons when reviewing imaging prior to making a definitive decision about surgical exploration. This guidance may be less relevant for some radiologists. Perhaps those radiologists with less experience with blunt trauma may find that scoring systems can aide in crafting an impression of the CT findings. The radiologists who participated in the development of the RAPTOR score, in fact, use the RAPTOR score in clinical practice [10] and we encourage providers to collaborate more closely with their radiology team Nonetheless, we are of the opinion that surgical judgment combined with radiologist overall impression of the scan precludes the need for a scoring system. Future research should be aimed at refining BIPS and RAPTOR scores.

This study has some limitations that are worthy of highlighting. This study involved patients with blunt abdominal trauma managed at a single regional trauma center. The radiologists who interpreted the associated CT scans are a single full-time academic practice group committed to our level 1 trauma center and have a considerable experience with the interpretation of scans performed for trauma. Trauma scans were routinely performed with trauma-specific protocols and were completed with the same CT technology over the course of the study. It is unlikely that these results would be reproducible from a group of radiologists who have a more limited or inconsistent experience with reading trauma CT scans. In addition, the confirmation of sBMI required that a patient underwent operation. The 90 patients who did not undergo an operation were observed for a median stay of 5.5 days. These patients had CT findings that represented bowel and/or mesenteric injury and were coded as such by the trauma registrars, but they never underwent laparotomy during their hospital stay. It is indeed possible that some of these patients may have had latent sBMI and required laparotomy following hospital discharge. We believe that this is an unlikely scenario as it is our perception that most patients either return to our center for complications or are transferred back to our center for continuity of care, and most latent bowel injuries present prior to 5 days of observation. Seven patients expired prior to surgery and could not be included in the analysis. Although this represents less than 5% of the sample, it is possible that results could be different given the inclusion of these patients. Lastly, this was a retrospective study and relied on the medical record to retrospectively determine components of the BIPS score and to determine sBMI from the operative report in the medical record. Ultimately, a high degree of suspicion for sBMI regardless of CT findings is warranted if clinical findings and mechanism of injury are consistent with possible bowel injury. As reported in this and prior studies, a “negative” CT scan cannot be entirely relied upon to rule out sBMI [15].

## Conclusion

This study is, to our knowledge, the first to evaluate the predictive utility of the radiologist impression of admission CT scan of the abdomen and pelvis in the setting of blunt abdominal trauma. We identified that the radiologist’s impression correlated well with sBMI, performed as well as two scoring rubrics (BIPS and RAPTOR), and may be used to direct treatment. Although surgical vigilance, including both surgeon evaluation of the CT images and close evaluation of the patient, remains fundamental to early diagnosis, the radiologist’s impression of the CT scan can be essential and perhaps helps simplify the approach to patients with possible sBMI.
